# OA06.04. Dose-response of spinal manipulation for chronic low back pain: pain and disability outcomes from a randomized controlled trial

**DOI:** 10.1186/1472-6882-12-S1-O24

**Published:** 2012-06-12

**Authors:** M Haas, D Vavrek, D Peterson

**Affiliations:** 1 Center for Outcomes Studies, University of Western States, Portland, USA

## Purpose

Report short- and long-term low back pain and disability outcomes from a randomized controlled trial on the quantity of visits to a chiropractor for nonspecific chronic low back pain (cLBP).

## Methods

We randomized 400 patients with cLBP to receive one of four dose levels of care: 0, 6, 12, or 18 sessions of lumbar spinal manipulation from a chiropractor. All participants were scheduled for three visits per week for six weeks at one of eight clinics. Care was rendered at each visit, either the index intervention or light massage control. The primary outcomes were low back pain intensity and functional disability at 12 and 24 weeks (100-point scales). Secondary outcomes included days with pain and functional disability, pain unpleasantness, global perceived improvement, and general health status. Outcomes were assessed at baseline and at 6, 12, 18, 24, 39, and 52 weeks. Linear dose effects and group comparisons (efficacy) were evaluated using covariate-adjusted simultaneous regression for continuous data (primary analysis). Binomial regression was performed in a responder analysis of 50% improvement (secondary analysis).

## Results

Adherence to all 18 treatment visits was 94%. Small linear dose-response effects were observed at 6 to 18 weeks and 52 weeks (p < .02). In comparison to the no-manipulation control, the optimal adjusted mean differences (MD) in the short term were found for 12 treatment sessions at 12 weeks: MDpain = 8.6 (3.2 to14.0) and MDdisability = 7.5 (1.7 to 13.3). No differences were found at 24 weeks. Differences at 52 weeks were maximized with 18 visits: MDpain = 7.6 (2.0 to13.2) and MDdisability = 8.8 (3.3 to 14.4). Responder analysis supported these results.

## Conclusion

Spinal manipulation visits had modest effects on cLBP outcomes. Best outcomes were for 12 visits in the short term (12 weeks) and 18 visits in the long term (52 weeks).

